# Toxicological Assessments of a Proprietary Blend of *Punica granatum* Fruit Rind and *Theobroma cacao* Seed Extracts: Acute, Subchronic, and Genetic Toxicity Studies

**DOI:** 10.1155/2022/3903943

**Published:** 2022-05-06

**Authors:** Ravi Kumar Madireddy, Krishnaraju Venkata Alluri, Venkateswarlu Somepalli, Trimurtulu Golakoti, Krishanu Sengupta

**Affiliations:** Laila Nutraceuticals R&D Center, Vijayawada, Andhra Pradesh, India

## Abstract

LN18178 (Tesnor^®^) is a standardized, proprietary composition of aqueous ethanol extracts of *Punica granatum* fruit rind and *Theobroma cacao* seeds. The present study demonstrates a broad-spectrum toxicological evaluation of LN18178 utilizing *in vitro* and *in vivo* preclinical models following the Organization for Economic Cooperation and Development (OECD) guidelines for testing chemicals. Wistar rats did not show any clinical signs of toxicity and morbidity in acute oral and dermal toxicity tests with the median lethal dose (LD_50_) values of at least 5000 mg/kg and 2000 mg/kg body weight, respectively. LN18178 was nonirritating to the skin and eyes of the treated rabbits. In a ninety-day subchronic repeated oral dose toxicity study, the LN18178-treated Wistar rats did not show dose-related signs of toxicity on their body weight, food consumption, organ weights, hematology, and clinical chemistry parameters. The estimated no-observed-adverse-effect level (NOAEL) of LN18178 in male and female rats was 2500 mg/kg body weight. The observations from the bacterial reverse mutation test, *in vitro* chromosomal aberration assay, micronucleus assay in mouse bone marrow erythrocytes, and *in vitro* mouse lymphoma TK^+/−^ gene mutation assay suggest that LN18178 is neither mutagenic nor clastogenic. In summary, the present study demonstrates that oral consumption of the herbal blend LN18178 does not show signs of toxicity; also it does not elicit genetic toxicity in the standard preclinical models.

## 1. Introduction

LN18178 is a proprietary combination of *Punica granatum* fruit rind and *Theobroma cacao* seeds extracts. The final formulation was standardized to contain at least 3.5% punicalagins and 0.5% theobromine. *Punica granatum* fruit or pomegranate rind and *Theobroma cacao* seeds or cocoa beans are popular ingredients in the confectionery and beverage industries worldwide [1, 2]. Pomegranate fruit rind has high nutritional value. It contains tannins, flavonols, anthocyanins, and phenolic and organic acids, having antioxidant, immunomodulatory, antidiabetic, antiplasmodial, antimicrobial, wound healing, antihyperglycemic, and hepatoprotective benefits [3, 4]. The cocoa seeds are rich in polyphenols; these dietary antioxidants provide several health benefits, including improved fat and carbohydrate metabolism, endothelial function, platelet function, and reduced inflammation [2, 5].

In a recent clinical study, LN18178-supplemented young male subjects have shown significant improvements in the serum testosterone level and muscle mass and strength. This fifty-six-day study demonstrated that LN18178-supplemented participants did not show any major adverse events. Their routine clinical chemistry parameters and vitals were within the normal range [6]. The study concluded that the herbal composition was tolerated well by the participants.

The objective of the present study was to evaluate a wide range of safety of LN18178 in the OECD-recommended *in vitro* and *in vivo* models. Here, we present a toxicological evaluation of LN18178 that includes acute oral toxicity, acute dermal toxicity, and ninety-day repeated dose toxicity studies in Wistar rats and acute dermal irritation and eye irritation studies in New Zealand albino rabbits. Further, the observations from a bacterial reverse mutation assay, *in vitro* chromosome aberration test, a micronucleus assay in mouse bone marrow erythrocytes, and an *in vitro* mammalian cell gene mutation assay using TK^+/−^ gene are also presented to demonstrate the genetic safety of LN18178.

## 2. Materials and Methods

### 2.1. Study Material

LN18178 (Tesnor^®^) is a standardized, proprietary composition of aqueous ethanol extracts of *Punica granatum* fruit rind (PG) and *Theobroma cacao* seed (TC), combined at a ratio of 4 : 1. LN18178 is standardized to contain at least 3.5% punicalagins and 0.5% theobromine, as measured using a high-performance liquid chromatographic (HPLC) method. The final product is formulated into a free-flowing powder containing 25% excipients. LN18178 is manufactured in a Good Manufacturing Practice (GMP)-certified facility of Laila Nutraceuticals, Vijayawada, India [6].

### 2.2. Plant Raw Materials and Extraction Procedures

The plant raw materials PG and TC were procured from Sangli, Maharashtra, and Aswaraopet, Telangana, India, respectively. The voucher specimens of the plant materials are preserved in the Taxonomy Division of Laila Nutraceuticals R&D Centre, Vijayawada, India. Typically, dried PG was pulverized to a coarse powder and extracted with aqueous ethanol at 55–60°C under continuous percolation. The extract was filtered and concentrated under a vacuum at 60–70°C to obtain *Punica granatum* fruit rind extract as a brown-colored powder. For TC, the dried raw material was pulverized to a coarse powder and extracted with aqueous ethanol at 55–60°C under continuous percolation. The extract was filtered and concentrated under a vacuum at 60–70°C to obtain *Theobroma cacao* seed extract as a brown-colored powder [6].

### 2.3. Cells, Chemicals, and Reagents

The *Salmonella typhimurium* strains (TA97a, TA98, TA100, TA102, TA1535, and TA1537), *Escherichia coli* strain (WP2uvrA), and lyophilized rat liver S9 fraction were purchased from Molecular Toxicology Inc., Boone, NC. Mouse lymphoma cells (L5178Y, clone-3.7.2c TK^+/−^), DMEM, and fetal bovine serum (FBS) were obtained from ATCC, Manassas, VA; Bacto agar was procured from Becton Dickinson (Sparks, Maryland). 2-Aminoanthracene, 2-nitrofluorene, sodium azide, 9-aminoacridine, 4-nitroquinoline-1-oxide, mitomycin C, cyclophosphamide, and colchicine were purchased from Sigma-Aldrich Corporation (St. Luis, MO). The clinical chemistry and hematology reagents were sourced from ILab Aries (Milano, Italy) and Mindray (Shenzhen, China). The analytical and laboratory reagents were purchased from Sigma-Aldrich Chemicals (Bengaluru, India).

### 2.4. Experimental Animals and Animal Husbandry

Specific pathogen-free animals were used in the present study. The Institutional Animal Ethics Committee (IAEC) of Laila Nutraceuticals, Vijayawada, India, approved the study protocols. Animal care and the experimental procedures were in compliance with the guidelines of the Committee for the Purpose of Control and Supervision of Experiments on Animals (CPCSEA), Government of India.

Male and female Wistar rats (6–10 weeks old) were purchased from Vivo Biotech Limited (Hyderabad, India). Six to eight-week-old Swiss albino mice and New Zealand white male rabbits (9–10 weeks, weighing 2.0–2.8 kg) were obtained from Palamur Biosciences Pvt. Ltd. and Mahaveera Enterprises, Hyderabad, India, respectively. Animals were acclimatized to the laboratory conditions for 5 to 7 days before the start of the experiment. The animals were housed in standard, individually ventilated cages, and the animal rooms were maintained at 21 ± 2°C, 40–70% relative humidity, with a 12-h light and dark cycle. Clean sterilized corn cob manufactured by Sparconn Life Sciences (Bengaluru, India) is used as the bedding material. Animals were allowed free access to standard diet and reverse osmosis (RO) water. The standard pellet diets for rabbits and rodents were procured from SDS Diet (Essex, UK) and Altromin (Lage, Germany). All studies followed the OECD (Organization for Economic Cooperation and Development) guidelines and were performed in a Good Laboratory Practice (GLP)-accredited facility at Laila Nutraceuticals Research and Development Center, Vijayawada, India, as described earlier [7].

### 2.5. Acute Oral and Dermal Toxicity Studies

An acute oral toxicity study in Wistar rats (8–9 weeks) was conducted following the OECD test guideline 425 [8]. Initially, LN18178 was orally administered to one male and one female rat at a dose of 5000 mg/kg body weight (BW). After LN18178 administration, the animals were closely monitored for clinical signs or morbidity/mortality up to 4 hr and daily for the next 14 days. The BW was measured weekly, and the rats were subjected to gross pathological examination following CO_2_ euthanasia on day 15. Further, to confirm the observation, two additional rats of each sex were gavaged with the same dose (5000 mg/kg BW) and followed a similar procedure.

An acute dermal toxicity study was performed following the OECD test guideline 402 [9]. A limit test was conducted at a dose level of 2000 mg/kg BW in one male and one female Wistar rat. Required quantity of LN18178 is soaked with water and applied to the shaved skin of the dorsal trunk of the animals. The application was protected with a bandage and allowed contact with the skin for 24 hr. The test item was removed, and the skin was gently cleaned with a water-soaked cotton swab. The animals were monitored for clinical signs and mortality/morbidity once a day for 14 days. On day 15, the animals were subjected to gross pathological examination following the CO_2_ euthanasia. Further, the test procedure at the same dose was repeated in an additional two male and two female rats in sequence with a time gap of 48 hr between the treatments.

Individual animal body weight (XS6002S precision balance, sensitivity 0.01 g; Mettler Toledo, India) was recorded before the test item administration/application on the day of dosing and weekly after that.

### 2.6. Skin and Eye Irritation Studies

The acute dermal irritation/corrosion study was done in healthy adult male New Zealand white rabbits following the OECD test guideline 404 [10]. Briefly, 0.5 g of moistened LN18178 was put on a gauge pad and applied to the shaved skin on the left dorsal flank of a single rabbit. The gauge pad was secured safely with adhesive tape and allowed for 4 hr. The pad was removed, and the signs of skin reactions were observed for up to 72 hr. The test procedure was repeated on two more rabbits. Animals were examined for signs of erythema and edema [11] at 60 min and then at 24, 48, and 72 hours after the test item removal.

An acute eye irritation study was performed to assess irritant/corrosive potential of LN18178 to the eyes of healthy young male New Zealand white rabbits following the OECD test guideline 405 [12]. A hundred milligram of LN18178 was instilled into the conjunctival sac of each rabbit's left (test) eye by gently pulling the lower lid away from the eyeball. The eyelids were held together for a few seconds to prevent the loss of the test item. After 1 hr, the treated eye was rinsed with sterile saline. The untreated right eye served as the control. The study used a topical anesthetic (0.5% Proparacaine HCl) and systemic analgesics (Tramadol 10 mg/kg and Meloxicam 0.5 mg/kg) to avoid or minimize pain and distress in the animals. The initial test was carried out using a single animal. The signs of eye irritation in the conjunctiva, iris, and cornea of both eyes were scored 1, 24, 48, and 72 hr after the test item removal using an ophthalmoscope [13]. The observation was confirmed by performing a similar test procedure on two more animals. Classification of eye irritation scores was determined with the maximum mean total score (MMTS) by the descriptive primary eye irritation scores system [14]. The animals were also observed for mortality/morbidity and clinical signs of toxicity during the observation period in the skin and eye irritation studies.

### 2.7. Subchronic (90-Day) Repeated Oral Toxicity Study

A ninety-day repeated dose oral toxicity study was conducted in young, healthy adult male and female Wistar rats following the OECD test guideline 408 [15]. The male and female rats were randomly assigned into four main groups (*n* = 20; ten males and ten females) and two reversal groups (*n* = 10; five males and five females). The rats in the main groups, namely, G1 (control), G2 (low), G3 (mid-dose), and G4 (high dose) received 0, 625, 1250, and 2500 mg/kg BW of LN18178, respectively. LN18178 prepared in 0.5% CMC-Na was administered daily through oral gavage using an intubation cannula. The G1 rats received only 0.5% CMC-Na through oral gavage daily for 90 days. On day 91, the animals in the main groups were euthanized by CO_2_ inhalation. The two reversal groups G1R and G4R received oral gavages of 0.5% CMC-Na and 2500 mg/kg BW LN18178, respectively, for ninety days and continued their in-life phase with regular rodent chow for an additional twenty-eight days, without the vehicle or LN18178 supplementation.

Animals were observed for morbidity, mortality, and clinical signs daily. The animals' body weight and feed consumption were recorded weekly once during the treatment and the reversal period. Ophthalmological examination was performed before the start of treatment, on day 91, and at the end of the reversal phase. Battery of functional observation (FOB) tests on the main group animals were conducted during the final week of the study.

Clinical pathology examination was performed at the end of the treatment period for the main group animals and the reversal period for the reversal group animals. Blood samples were collected from the heart under isoflurane anesthesia. The rats fasted overnight before the blood collection. Hematological parameters were analyzed from K_2_-EDTA blood using a BC-5000 Vet hematology analyzer (Mindray^®^, Shenzhen, China) except the reticulocyte count and clotting time estimated by the manual slide reading and capillary tube method, respectively.

The hematology parameters included red blood cell count (RBC), white blood cell count (WBC), neutrophils (Neu), lymphocytes (Lym), eosinophils (Eos), monocytes (Mono), basophils (Baso), platelets (Plt), reticulocytes (Rec), hemoglobin concentration (Hb), hematocrit (Hct), mean corpuscular volume (MCV), mean corpuscular hemoglobin (MCH), and mean corpuscular hemoglobin concentration (MCHC).

Clinical biochemistry parameters in the plasma samples were measured using a fully automated clinical chemistry analyzer (I Lab Aries^®^; I Lab, Bedford, MA, USA). The biochemical parameters included glucose (Glu), blood urea nitrogen (BUN), urea (Ur), total cholesterol (T.Chol), triglycerides (Trig), total bilirubin (T.Bil), aspartate aminotransferase (AST), alanine aminotransferase (ALT), alkaline phosphatase (ALP), total protein (TP), albumin (Alb), calcium (Ca), phosphorus (Phos), sodium (Na), potassium (K), and chloride (Cl). The electrolytes, i.e., sodium (Na), potassium (K), and chloride (Cl), were analyzed using an electrolyte analyzer (I Lyte®; I Lab, Bedford, MA, USA).

Estrous cycle examination was performed by observing the cellular pattern of vaginal smear for main and recovery group female animals on the day of necropsy.

Total thyronine or triiodothyronine (T3), thyroxin (T4), and thyroid-stimulating hormone (TSH) in the serum samples were measured using commercially available enzyme immunoassay (EIA) kits. T3 and T4 kits were procured from Calbiotech, USA; and the TSH kit was procured from BioVendor, Czech Republic. The assays were performed following the procedures provided in the user manuals. The assay plates were read in a microplate reader (X-Mark; Bio-Rad Laboratories, Hercules, CA, USA).

At the end of the observation period, the animals from the main and the reversal groups were euthanized and subjected to gross pathological examination. The overnight-fasted animals were euthanized with carbon dioxide inhalation. All animals and their excised organs were macroscopically examined. The collected organs were the liver, kidneys, heart, lungs, brain, spleen, adrenal glands, thymus, testes, epididymis with seminal vesicles (males), ovaries, and uterus with the cervix (females). The organs were weighed on an electronic balance with 0.01 g accuracy (Mettler Toledo, Columbus, OH).

Tissues and organs collected from the control (G1) and high-dose (G4) groups were processed for a histopathology examination. Small pieces of the tissues were fixed in 10% neutral buffered formaldehyde for 48 hr, dehydrated with alcohol, embedded in paraffin, and cut into 3–5 *μ*m-thick sections using a rotary microtome (Leica Biosystems, Nussloch, Germany). The tissue sections were processed in graded alcohol and stained with hematoxylin and eosin. The stained tissue sections were examined under a light microscope (Axio Scope; Carl Zeiss, Munich, Germany).

### 2.8. Genotoxicity Studies

#### 2.8.1. Bacterial Reverse Mutation Assay

The ability of LN18178 to induce the point mutations was evaluated in the histidine gene locus of *Salmonella typhimurium* and tryptophan gene locus of *Escherichia coli* WP2uvrA in the presence or absence of the metabolic activation system S9 using the plate incorporation method following the OECD test guideline 471 [16]. The bacterial strains were exposed to LN18178 at concentrations of 312.5, 625, 1250, 2500, and 5000 *μ*g/plate. The test plates were incubated for 48–72 hr at 37°C. The vehicle control culture plates contained 1% DMSO mixed with the bacterial culture broth. In the test plates, the number of revertant colonies was counted manually. The cultures in the presence of the strain-specific mutagens 2-aminoanthracene, 2-nitrofluorene, sodium azide, 9-aminoacridine, or 4-nitroquinoline-1-oxide were the positive controls.

#### 2.8.2. *In Vitro* Chromosome Aberration Assay

The clastogenic effect of LN18178 was evaluated in human peripheral blood lymphocytes following the OECD test guideline 473 [17]. Briefly, peripheral blood lymphocytes were obtained by venipuncture from a healthy human male volunteer of 29 years with no history of smoking, illness, viral infection, or recent exposure to drugs/radiation. The Alluri Sitarama Raju Institutional Ethics Committee, Eluru, Andhra Pradesh, India, reviewed and approved the study procedures. The highest concentration of the test item was determined in a cytotoxicity assay conducted on the isolated lymphocytes. The cells were treated with different concentrations of LN18178 and incubated for 24 hr. The cells in the vehicle control wells were treated with 1% DMSO. Solubility of LN18178 and drastic pH change of the culture medium were carefully observed in the treated wells. The cytotoxicity observations selected a dose range of 250 and 2000 *μ*g/mL LN18178 in the chromosomal assay. The cells were treated in the presence or absence of an S9 metabolic activator for up to 22 hr. Mitomycin C or cyclophosphamide was treated in the positive control wells, and 1% DMSO was added in the negative or vehicle control wells. Three hundred cells in each treatment well were scored for the presence of structural chromosomal aberration.

#### 2.8.3. *In Vivo* Micronucleus Assay

The micronucleus assay was performed on the bone marrow erythrocytes of Swiss albino mice following the OECD test guideline 474 [18]. Briefly, fifty (25 males and 25 females) mice were equally distributed into five groups, i.e., G1—0.5% CMC-Na as vehicle control; G2, G3, G4—500, 1000, 2000 mg/kg·BW LN18178 mixed in 0.5% CMC-Na, respectively. The test item or the vehicle was administered via oral gavage two times at an interval of 24 hr. The positive control group or G5 rats received 40 mg/kg·BW cyclophosphamide monohydrate through intraperitoneal (i.p.) injection only once on the second day, 22 hr before euthanasia. All animals were examined for clinical signs and were euthanized by CO_2_ asphyxiation, and bone marrow samples were aspirated from the femur bones. The bone marrow samples were smeared on glass microscope slides, and the smears were stained with 6% Giemsa following the standard procedure. The stained bone marrow samples were examined under a 20x objective lens of a light microscope (ECLIPSE E200; Nikon Corporation, Tokyo, Japan). In each sample, 4000 polychromatic erythrocytes (PCEs) were scored. The frequency of micronucleated polychromatic erythrocytes (MNPCEs) is expressed as a percentage.

#### 2.8.4. *In Vitro* Mouse Lymphoma L5178Y (Tk^+/−^) Forward Mutation Assay

Mouse lymphoma assay was performed to ascertain the potential mutagenic effect of LN18178 to induce forward mutations in the TK locus of the L5178Y TK^+/−^ mouse lymphoma cell line [19]. In the preliminary cytotoxicity test, L5178Y/TK^+/−^ cells were exposed to vehicle control (1% DMSO) and six concentrations of test article, the highest concentration being the lowest insoluble dose in the treatment medium (2000 *μ*g/mL). The cytotoxicity was measured by assessment of the relative total growth (RTG) of the treated cultures to the vehicle control cultures. The RTG measure considers all cell growth/loss during treatment and a two-day expression period (RSG).

The mammalian mutation assay was performed by treating the duplicate cultures of L5178Y/TK^+/−^ cells with 500, 250, 125, and 62.5 *μ*g/mL LN18178 in the presence or absence of metabolic activation for 3–4 hr and 125, 62.5, 31.25, and 15.6 *μ*g/mL LN18178 in the absence of metabolic activation for 24 hr. The maximum doses showed 10–20% RTG in the cytotoxicity assays. The positive, negative, or vehicle controls were run in duplicates, parallel to LN18178 treatments. Briefly, 6 × 10^6^ cells per culture (sample) were treated in DMEM supplemented with 10% FBS and incubated at 37°C in a humidified atmosphere with 5% CO_2_. After the treatment period, the cultures were washed and counted, and 2 × l0^5^ cells/mL were subcultured for 48 hr to express induced mutations (Tk-deficient phenotype). At the end of the expression period, for mutant frequency (MF) determination, 2000 cells were seeded in each well of a 96-well plate containing a medium with the selective agent (TFT). In a separate 96-well plate, the cell viability (V) was determined by seeding an average of 1.6 cells in each well containing the media without the selective agent. The plates were incubated at 37°C with 5% CO_2_ for 10–14 days. Subsequently, the wells were examined for counting the colonies with the help of adding an MTT solution (2.5 mg/mL). The mutant frequency of LN18178 was calculated using the number of colonies formed on the TFT-selection plates divided by the cloning efficiency of the nonselection plates, which is expressed as the number of mutants per million viable cells. Small colony mutant frequency was calculated using the relevant number of empty wells for small (size ˂ than ¼ diameter of the well) colonies. The colony size was estimated following the method described by Honma et al. [20].

### 2.9. Statistical Analysis

The data were presented as mean ± SD. The data were analyzed using GraphPad Prism software version 8.0.2 (GraphPad Software, La Jolla, CA). In the main groups, “between the groups” comparison analyses were performed using the one-way analysis of variance (ANOVA) test and Student's *t*-test in the reversal groups. All comparisons were evaluated at the 95% level of confidence, and the *p*-values less than 0.05 were considered statistically significant.

## 3. Results

### 3.1. Acute Oral and Dermal Toxicity

In the acute oral and dermal toxicity tests, LN18178 did not produce any signs of toxicity or mortality in the 14-day observation period of the studies. All animals survived till they were humanely sacrificed. The animals gained their normal body weight during the study period. In gross necropsy, LN18178-treated animals did not show pathological changes in the vital organs.

### 3.2. Acute Dermal and Eye Irritation

In the acute dermal irritation study, none of the animals showed LN18178 treatment-related dermal reactions up to 72 hr of the patch removal. The mean skin reaction for three rabbits was scored at 24, 48, and 72 hr of observations, and the average score was 0.00 for erythema and edema.

The acute eye irritation study did not show treatment-related significant clinical signs or mortalities in the experimental animals. After removing the LN18178 application, conjunctival redness was observed until 48 hr with no signs of irritation to the cornea and iris. Chemosis was also observed at 24 hr and normalized after 48 hr of application. Together, the observations suggest that LN18178 is considered as “nonirritant” to the skin and the eyes of New Zealand white rabbits.

### 3.3. Subchronic (90-Day) Repeated Dose Oral Toxicity Study

Ninety days of oral administration of LN18178 did not show any clinical signs of toxicity and mortality in male and female rats. The animals in the main groups, including the reversal groups, were healthy and active. All animals in the main and reversal groups survived through the end of the study. Ophthalmic observations did not reveal any treatment-related changes in the treated rats at the end of the experimental period. At the end of the study, the LN18178-treated male and female rats did not show any changes in the neurobehavioral assessments that included visual response, auditory response, gait, landing foot splay, grip strength, and locomotor activity, compared with the control rats.

#### 3.3.1. Body Weight and Food Consumption

Table 1 presents the bodyweight growth pattern of the male and female rats during the study. LN18178-administered rats in the main groups, including the reversal groups, did not show treatment-related changes in their overall body weight compared with the vehicle control rats. All groups of LN18178-supplemented male and female rats showed a regular pattern of bodyweight gain consistent with their ages as observed in the sex-matched control rats during the study (Table 1). Compared with the control rats, the food consumption by the LN18178-treated rats in the main and reversal groups was not statistically different (Table 2).

#### 3.3.2. Hematological Parameters

In the majority, LN18178 administration did not produce any treatment-related changes in the hematological measures (Table 3). However, in the main groups, a few significant changes include a lower RBC count, increased MCH and MCHC in 625 mg/kg male rats, decreased WBC, increased eosinophil count in 1250 mg/kg male, and reduced platelet count in 2500 mg/kg male rats. In the 2500 mg/kg male reversal group, increased numbers of neutrophils and platelets and decreased MCV were observed compared with the control rats. In female rats of the main groups, 1250 mg/kg rats showed increased MCH. Compared with the control rats, the high-dose reversal female rats showed decreased lymphocytes and hematocrit and increased eosinophils, MCHC, and platelets (Table 3). However, the changes of these indices in the LN18178-treated male and female rats were random, not dose-dependent.

#### 3.3.3. Serum Biochemical Parameters

Table 4 presents the clinical chemistry parameters in serum samples of the LN18178-treated male and female rats. Except for a few parameters, LN18178 treatment did not show significant changes in the clinical chemistry parameters compared with the control rats. In the main groups, male rats in 2500 mg/kg and 1250 mg/kg showed decreased ALT and increased calcium, respectively. Compared with the control, creatinine and phosphorus were reduced in the reversal male rats. The measurements in the female rats of the main and reversal groups are not statistically different compared with the controls (Table 4).

#### 3.3.4. Necropsy and Organ Weights

Necropsy of the treated animals showed normal appearance of the major organs and tissues of the male and female rats (Tables 5 and 6, respectively). Absolute and relative organ weights of treated animals were comparable with their respective control groups' with no significant changes, except 50% reductions in the combined weight of thyroid and parathyroid glands of the mid- and high-dose male rats (Table 5) and an 18.18% reduction of the relative weight of the spleen in the high-dose female rats compared with control rats (Table 6).

#### 3.3.5. Hormone Analysis

Oral administration of LN18178 for ninety days did not yield significant changes in T3, T4, and TSH concentrations in the rats of the main and reversal groups when compared with respective control groups (Table 7).

#### 3.3.6. Macroscopic and Microscopic Examinations

The gross morphology of the vital organs of the high-dose male and female rats was unaltered following the LN18178 treatment and reversal period. Overall, the treatment-related effects on the main and reversal groups' vital organs were no different from those of the sex-matched controls.  Table 8 summarizes the histological observations on the vital organs of the male and female rats. In the microscopic examination, the liver showed normal hepatocytes, absence of any lesions; in the kidneys, normal glomeruli with Bowman's capsule were present with no lesion. The spleen showed normal lymphoid follicles; the brain exhibited the cerebral cortex's normal appearance. The ovaries showed normal cyclic patterns with different stages of growing follicles, blood vessels, and stromal cells. Testes of only one male rat in the high-dose main group showed inflammatory changes in the epithelial cells of the seminiferous tubule; however, no such changes were observed in the reversal male rats (data not shown). The findings on the remaining organs or tissues were consistent with the control rats (Table 8).

### 3.4. Bacterial Reverse Mutation Assay (Ames Test)

LN18178 showed no mutagenicity up to 5 mg/plate when tested on *S. typhimurium* strains TA97a, TA98, TA100, TA102, TA1535, and TA1537 and *E. coli* WP2uvrA in the absence or presence of metabolic activation. The positive control reference plates treated with the strain-specific mutagens significantly increased the number of revertant colonies (Table 9).

### 3.5. *In Vitro* Chromosome Aberration Test

The chromosome aberration test was conducted to identify whether LN18178 treatment results in any chromosomal abnormalities. The observations indicate that LN18178 treatment did not significantly alter the percentage of structural chromosome aberrations and polyploidy compared with the negative or vehicle control cells, with or without S9 metabolic activator. As expected, mitomycin C or cyclophosphamide treatments as the positive controls significantly increased the number of cells with the structurally abnormal chromosome in the presence or absence of the S9 metabolic activation (Table 10).

### 3.6. *In Vivo* Micronucleus Assay

In the bone marrow erythrocyte micronucleus assay, LN18178 supplementation did not yield significant changes in the ratio of polychromatic erythrocytes (PCE) and total erythrocytes (TE) compared with the control mice (Table 11). Also, LN18178 did not increase the frequency of micronucleated PCEs (MNPCEs) compared with the control mice. Cyclophosphamide treatment significantly increased the MNPCE frequency in the bone marrow samples compared with the vehicle control (Table 11).

### 3.7. *In Vitro* Mouse Lymphoma L5178Y (Tk^+/−^) Forward Mutation Assay

Based on the results obtained in the range-finding study, the treatment of cultures with LN18178 resulted in cytotoxicity observed as dose-dependent reductions in percent RTG in the treatment conditions (3 hr, +/−S9; and 24 hr, −S9) (Table 12). In mutagenicity assay, the mutant frequencies in the LN18178-treated cultures did not meet the global evaluation factor (GEF) for considering as a positive response. The positive controls, nitroquinoline oxide, and cyclophosphamide induced significant increases in mutant frequencies compared with concurrent control cultures (Table 12).

## 4. Discussion

The present study demonstrates a broad safety profile of an herbal composition, LN18178, a standardized, proprietary composition of aqueous ethanol extracts of *Punica granatum* fruit rind and *Theobroma cacao* seeds. The cocoa bean is an essential raw material for the confectionery and beverage industry, and pomegranate fruit rind is a popular ingredient used in dairy products [21, 22]. These two plant materials are rich in pharmacologically active several phytochemicals [23, 24], and their preparations are used to manage several ailments in traditional and alternative medicine [25, 26]. In general, medicinal plant-derived products are considered natural, and a long usage history defines their safety. Although a dietary supplement of herbal origin is generally considered safer than an allopathic drug, it is important to assess the safety profile of the herbal product in a battery of toxicological studies as recommended by the European Food Safety Authority (EFSA) and United States Food and Drug Administration (US-FDA) [27, 28]. To this objective, the present study was aimed to assess possible systemic and genetic toxicity of LN18178 in a wide range of studies in the recommended preclinical models following the OECD guidelines.

In the present investigation, the acute oral and dermal toxicity studies in Wistar rats suggest that the LD_50_ values of LN18178 are at least 5000 and 2000 mg/kg BW, respectively. LN18178 treatment did not show any acute toxicity or mortality or abnormalities in hematobiochemical parameters; the gross pathology of the vital organs was also unaltered. According to the numeric cut-off criteria set by the Globally Harmonized System (GHS), the substances with oral and dermal LD_50_ values greater than 2000–5000 mg/kg are categorized as unclassified or category 5 [29]. The study findings suggest that LN18178 has a relatively low acute toxicity hazard and falls into category 5. Also, in the dermal and eye irritation studies, LN18178 did not show signs of gross toxicity, irritation, or pathological changes. All animals appeared active and healthy. Based on the Harmonized Integrated Classification System for Human Health and Environmental Hazards of Chemical Substances and Mixtures, the observations for the present study conclude that LN18178 is not irritating to the eyes and skin [11, 14, 29]. The present subchronic toxicity study demonstrates that repeated oral treatment with LN18178 up to 2500 mg/kg BW for 90 days followed by a twenty-eight-day reversal phase did not yield significant clinical signs, behavior, body weight, or food consumption in the male and female rats, compared with the sex-matched controls. No animal in the primary or the reversal groups died during the study. Toxic chemical substances adversely impact the food intake and the natural growth of the experimental animals [30]. In the present subchronic study, the animals gained their natural body weight and consumed food consistent with their age and sex. Further, LN18178-treated male and female rats did not show any changes in the functional observational battery (FOB), a noninvasive tool that measures the neurobehavioral changes to assess the central nervous system (CNS) safety [31]. Together, these observations suggest that oral administration of LN18178 neither affected the healthy growth nor exerted a neurotoxic effect on the experimental rats.

In a fifty-six-day randomized, double-blind, placebo-controlled clinical study, LN18178 enhanced the serum levels of free and total testosterone in young male participants [6]. Therefore, it is important to know the effect of LN18178 treatment on the estrous cycle in female rats. In the present study, LN18178-treated female rats in the main and reversal groups did not show significant alterations in the frequency of the phases of their estrous cycle compared with the control rats. Also, the LN18178-treated rats did not show treatment-related changes in absolute and relative weights of the uterus and ovaries. Hence, LN18178 appears to be not toxic to the female reproductive organs of the experimental animals.

LN18178-treated male and female rats did not show significant treatment-related changes in their blood parameters compared with the untreated control rats. The blood parameters in the LN18178-treated rats of the main and reversal groups are comparable with the sex-matched control rats, with a few exceptions. The changes (increase or decrease) in parameters are within the limits of normal biological variations depending on the sex and age of the animals [32]. Also, these changes are not dose-related, except the platelet count in the main male rats. Although the male rats in the main groups showed a dose-dependent reduction in platelet count, the clotting time (CT) value was no different from that of the control group; the platelet count in the reversal male rats was comparable with the control group. Overall, these observations indicate that LN18178 had no toxic effects on the circulating blood cells or interfered with the hematopoiesis and the other blood cell formation.

The serum clinical chemistry analysis is important to evaluate a substance for its toxic effect on the liver and renal functions [33]. In the present study, the clinical chemistry parameters were within the normal range in the LN18178-treated rats of the main and reversal groups. However, a few parameters showed significant changes from the concurrent control rats. These changes are not related to the dose or the duration of the treatment. Elevated levels of the liver transaminases beyond the normal ranges indicate the liver injury or its abnormal function due to the toxic effect of the test item [34]. AST and ALT data and unaltered histological findings of the liver tissue suggest that LN18178 treatment did not yield a toxic effect on the normal function and structure of the liver of the experimental rats.

Further, unaltered levels of serum urea, creatinine, BUN, and electrolytes in the LN18178-treated rats suggest that the oral administration of the herbal composition had no adverse effect on the normal kidney function in the experimental rats [35]. Similarly, no changes in LDL, HDL, triglyceride, and glucose levels indicate the normal lipid and carbohydrate metabolism in the LN18178-treated rats. The absolute and relative weights of the combined thyroid and parathyroid glands were significantly reduced in the mid- and high-dose supplemented male rats of the main groups. The absolute weights of the combined thyroid and parathyroid glands are within the range of historical control data (0.0134 to 0.690 g). However, there were no treatment-related changes in serum T3, T4, and TSH levels and histological changes of the thyroid-parathyroid tissue in the experimental rats of these groups. In addition, the carbohydrate and lipid metabolism markers did not alter in the treated rats. Therefore, the thyroid-parathyroid tissue weight changes are not treatment-related and not considered toxicologically relevant. These data suggest that oral administration of LN18178 did not impact the endocrine function of the thyroid on lipid metabolism [15]. Overall, LN18178 supplementation did not affect the vital organs' normal function and did not cause abnormal metabolic changes in the rats.

No treatment-related morphological changes were observed in the gross and microscopic examinations of the vital organs of the LN18178-treated rats. These observations further support that ninety days of oral administration of LN18178 did not cause systemic toxicity in the male and female rats. Taken together, the results of the present subchronic repeated dose oral toxicity study establish that the no-observed-adverse-effect level (NOAEL) of LN18178 in the male and female rats is 2500 mg/kg BW per day, which is greater than 26 g of daily human dose, considering an average BW of sixty-five kg. This estimated human equivalent dose is at least 65-fold higher than the tested dose in the human study [6].

Further, the *in vitro* and *in vivo* genotoxicity studies demonstrate that LN18178 is neither mutagenic nor clastogenic, suggesting LN18178 does not cause DNA or chromosomal damage.

## 5. Conclusions

In conclusion, the present toxicity studies suggest the proprietary composition LN18178 (Tesnor^®^) has a broad range of safety profiles. LN18178 has no genotoxic potential and no systemic toxicity through oral administration. The NOAEL of LN18178 from the 90-day repeated oral dose toxicity study is 2500 mg/kg·BW/day. Together, the observations from the present study raise no toxicity concern and affirm a broad-spectrum safety of LN18178.

## Figures and Tables

**Table 1:** Effect of 90-day oral administration of LN18178 on the body weights of male and female Wistar rats.

Day	Sex	LN18178 dose (mg/kg·BW)					
		Main groups				Reversal groups	
		0	625	1250	2500	0	2500
Predose	M	166.75 ± 12.55	171.25 ± 16.34	168.01 ± 9.50	167.91 ± 17.14	165.86 ± 11.6	163.74 ± 7.18
	F	141.19 ± 8.15	141.64 ± 8.33	140.57 ± 6.03	142.17 ± 7.23	136.2 ± 8.81	134.97 ± 10.37
28	M	285.94 ± 23.68	290.09 ± 19.01	294.93 ± 31.15	287.31 ± 26.16	300.11 ± 23.12	308.34 ± 17.42
	F	194.84 ± 12.16	193.16 ± 13.54	199.09 ± 11.19	194.63 ± 17.4	197.15 ± 7.31	196.44 ± 14.66
56	M	343.72 ± 38.66	342.87 ± 24.01	345.63 ± 39.79	337.59 ± 32.12	354.96 ± 30.64	351.76 ± 24.27
	F	213.90 ± 13.90	210.21 ± 15.19	219.31 ± 16.75	212.69 ± 23.13	217.68 ± 10.03	214.27 ± 21.31
84	M	372.66 ± 44.86	375.14 ± 23.28	374.66 ± 42.23	373.25 ± 33.77	386.13 ± 32.16	382.56 ± 22.64
	F	228.78 ± 12.98	225.45 ± 19.02	227.51 ± 16.12	226.53 ± 23.91	232.27 ± 14.31	231.7 ± 17.23
91	M	379.28 ± 46.31	382.28 ± 23.81	382.19 ± 40.82	377.74 ± 32.30	389.51 ± 31.39	384.88 ± 23.5
	F	230.63 ± 13.99	227.04 ± 18.53	229.14 ± 15.68	228.45 ± 23.48	235.37 ± 14.41	233.55 ± 16.62
118	M	—	—	—	—	417.73 ± 33.09	412.2 ± 31.23
	F	—	—	—	—	248.04 ± 13.36	245.94 ± 7.51

Data are presented as mean ± SD of body weight (BW) in *g*. *n* = 20 in main groups (10 males and 10 females) and *n* = 10 in reversal groups (5 males and 5 females).

**Table 2:** Effect of 90-day oral administration of LN18178 on food consumption by the male and female Wistar rats.

Day	Sex	LN18178 dose (mg/kg BW)					
		Main groups (*n* = 10)				Reversal groups (*n* = 5)	
		0	625	1250	2500	0	2500
1–28	M	569.72 ± 11.22	561.58 ± 32.82	567.08 ± 20.37	557.12 ± 16.76	583.35 ± 29.81	600.59 ± 48.34
	F	409.25 ± 20.98	397.91 ± 34.70	424.70 ± 22.53	408.33 ± 36.03	406.94 ± 1.97	401.87 ± 3.30
29–56	M	573.96 ± 7.68	561.10 ± 17.92	572.30 ± 14.53	579.39 ± 24.80	585.00 ± 40.78	600.59 ± 3.27
	F	397.95 ± 15.95	384.30 ± 25.07	406.68 ± 14.75	405.36 ± 35.61	402.66 ± 8.04	393.76 ± 5.39
57–90	M	664.16 ± 11.12	658.03 ± 17.16	658.05 ± 14.96	655.55 ± 39.59	700.68 ± 17.47	702.07 ± 21.83
	F	475.25 ± 20.07	473.43 ± 31.39	477.55 ± 10.56	478.88 ± 27.76	497.61 ± 9.28	489.33 ± 10.70
91–118	M	—	—	—	—	538.18 ± 10.91	528.25 ± 11.73
	F	—	—	—	—	370.78 ± 16.49	373.26 ± 33.81

Data are presented as mean ± SD of food consumption in *g*. Each main group contains 20 rats (10 males and 10 females), and reversal group contains 10 rats (5 males and 5 females). BW, body weight; M, male; F, female.

**Table 3:** Effect of oral administration of LN18178 on blood parameters in male and female Wistar rats.

Parameters	Sex	LN18178 Dose (mg/kg·BW)					
		Main groups				Reversal groups	
		0	625	1250	2500	0	2500
WBC (10^3^ cells/*μ*L)	M	9.22 ± 1.43	8.17 ± 1.86	7.32 ± 1.25^*∗*^	7.48 ± 1	8.42 ± 0.08	8.05 ± 1.23
	F	5.41 ± 1.17	6.05 ± 1.22	5.65 ± 0.89	5.15 ± 1.1	6.38 ± 1.39	6.78 ± 0.84
Neutrophils (%)	M	15.15 ± 2.82	13.58 ± 5.12	13.39 ± 3.76	14.69 ± 3.81	14.28 ± 0.58	15.2 ± 0.56^*∗*^
	F	12.14 ± 1.21	14.2 ± 5.69	14.13 ± 2.81	12.35 ± 2.42	11.5 ± 2.17	13.48 ± 0.55
Lymphocytes (%)	M	81.94 ± 2.94	83.11 ± 5.87	82.07 ± 5.03	81.53 ± 3.05	82 ± 0.82	81.14 ± 1.38
	F	84.73 ± 1.74	82.58 ± 6.43	82.44 ± 3.35	83.2 ± 2.68	84.96 ± 1.86	82.22 ± 0.96^*∗*^
Monocytes (%)	M	2.17 ± 1.07	2.38 ± 1.02	3.21 ± 1.52	2.67 ± 1.47	2.58 ± 0.43	2.58 ± 0.78
	F	2.26 ± 1.04	2.3 ± 0.77	2.55 ± 1.49	3.54 ± 1.45	2.5 ± 0.88	2.7 ± 0.45
Eosinophils (%)	M	0.74 ± 0.37	0.93 ± 0.25	1.33 ± 0.54^*∗*^	1.11 ± 0.38	1.14 ± 0.23	1.08 ± 0.68
	F	0.87 ± 0.33	0.92 ± 0.18	0.88 ± 0.29	0.91 ± 0.34	1.04 ± 0.15	1.6 ± 0.38^*∗*^
Basophils (%)	M	0 ± 0	0 ± 0	0 ± 0	0 ± 0	0 ± 0	0 ± 0
	F	0 ± 0	0 ± 0	0 ± 0	0 ± 0	0 ± 0	0 ± 0
RBC (10^6^ cells/*μ*L)	M	9.39 ± 0.66	8.76 ± 0.58^*∗*^	9.02 ± 0.39	9.04 ± 0.44	8.79 ± 0.19	8.84 ± 0.26
	F	8.49 ± 0.52	8.45 ± 0.39	8.16 ± 0.37	8.14 ± 0.38	8.37 ± 0.32	7.69 ± 0.67
Hemoglobin (g/dL)	M	16.9 ± 0.46	16.76 ± 0.71	16.71 ± 0.39	16.83 ± 0.6	16.62 ± 0.16	16.3 ± 0.78
	F	15.58 ± 0.52	15.8 ± 1.08	16.06 ± 0.48	15.78 ± 0.4	16.28 ± 0.62	15.28 ± 0.94
Hematocrit (%)	M	50.44 ± 2.09	48.95 ± 2.74	49.53 ± 1.62	49.7 ± 2.28	48.76 ± 1.09	47.5 ± 2.34
	F	46.9 ± 1.63	46.52 ± 2.48	47.31 ± 2.32	46.81 ± 1.99	48.46 ± 1.84	44.64 ± 2.51^*∗*^
MCV (fL)	M	53.9 ± 2.7	55.97 ± 2.06	54.95 ± 1.69	54.99 ± 1.34	55.44 ± 0.11	53.68 ± 1.52^*∗*^
	F	55.43 ± 3.64	55.07 ± 3.16	58.04 ± 2.59	57.55 ± 2.24	57.92 ± 0.23	58.24 ± 1.74
MCH (pg)	M	18.07 ± 0.98	19.19 ± 0.8^*∗*^	18.52 ± 0.47	18.63 ± 0.33	18.94 ± 0.34	18.42 ± 0.66
	F	18.44 ± 1.14	18.7 ± 1.37	19.72 ± 0.99^*∗*^	19.42 ± 0.99	19.48 ± 0.26	19.92 ± 0.44
MCHC (g/dL)	M	33.52 ± 0.58	34.27 ± 0.63^*∗*^	33.73 ± 0.64	33.9 ± 0.76	34.12 ± 0.59	34.3 ± 0.52
	F	33.24 ± 0.39	33.93 ± 0.62	33.99 ± 1.04	33.74 ± 1.45	33.6 ± 0.42	34.2 ± 0.32^*∗*^
Platelets (10^3^ cells/*μ*L)	M	825.5 ± 114.7	758.3 ± 82.6	741.7 ± 78.2	689.8 ± 50.9^*∗*^	722.2 ± 15.7	808.2 ± 68.6^*∗*^
	F	876.9 ± 96.6	833.2 ± 71.2	828.5 ± 65.5	879.8 ± 74.1	797 ± 74.2	950 ± 34.1^*∗*^
Reticulocytes (%)	M	1.13 ± 0.22	1.35 ± 0.22	1.2 ± 0.22	1.35 ± 0.23	1.2 ± 0.28	1.28 ± 0.19
	F	1.2 ± 0.29	1.29 ± 0.29	1.29 ± 0.31	1.36 ± 0.26	1.26 ± 0.27	1.14 ± 0.21
Clotting time (sec)	M	144 ± 23.66	150 ± 20	159 ± 20.25	153 ± 22.14	150 ± 21.21	156 ± 25.1
	F	147 ± 22.14	150 ± 24.49	150 ± 20	153 ± 26.27	132 ± 16.43	150 ± 21.21

Data are presented as mean ± SD; ^*∗*^ indicates significant changes (*p* < 0.05) compared with the control group. MCV, mean corpuscular volume; MCH, mean corpuscular hemoglobin; MCHC, mean corpuscular hemoglobin concentration; RBC, red blood cells; WBC, white blood cells.

**Table 4:** Effect of 90-day oral administration of LN18178 on clinical biochemistry parameters of male and female Wistar rats.

Parameters	Sex	LN18178 dose (mg/kg·BW)					
		Main groups				Reversal groups	
		0	625	1250	2500	0	2500
ALP (U/L)	M	85.5 ± 15.01	89.4 ± 22.15	81.4 ± 17.32	83.9 ± 17.62	88.4 ± 19.97	76.8 ± 9.5
	F	43.3 ± 8.65	45.1 ± 6.64	46.7 ± 8.83	45.1 ± 13.8	38 ± 13.08	39.4 ± 14.29
ALT (U/L)	M	62 ± 11.84	61.9 ± 8.94	57.8 ± 14.44	48.6 ± 11.43^*∗*^	56.4 ± 2.7	59.8 ± 6.14
	F	51.5 ± 7.82	50.1 ± 6.17	41.5 ± 10.06	52 ± 23.49	60.2 ± 6.83	55.6 ± 5.77
AST (U/L)	M	120.5 ± 22.8	108.3 ± 16.66	113.2 ± 26.2	108.9 ± 14.84	108 ± 13.25	109.2 ± 11.69
	F	102.8 ± 20.8	103.8 ± 21.4	93.3 ± 22.8	106.1 ± 15.02	111.6 ± 12.6	110.8 ± 16.9
T.Bil (mg/dL)	M	0.17 ± 0.16	0.13 ± 0.05	0.14 ± 0.05	0.18 ± 0.13	0.1 ± 0	0.1 ± 0
	F	0.13 ± 0.05	0.1 ± 0	0.12 ± 0.04	0.13 ± 0.05	0.1 ± 0	0.1 ± 0
BUN (mg/dL)	M	22.7 ± 3.47	23.7 ± 3.65	23.5 ± 4.35	21.7 ± 5.17	22 ± 2.45	20 ± 2.12
	F	21.9 ± 1.29	21.1 ± 2.28	20.5 ± 1.72	21.1 ± 3.78	20.8 ± 3.56	22.2 ± 1.3
Calcium (mg/dL)	M	9.16 ± 0.3	9.26 ± 0.22	9.66 ± 0.38^*∗*^	9.5 ± 0.41	10.06 ± 0.19	9.86 ± 0.17
	F	9.08 ± 0.26	9.33 ± 0.21	9.38 ± 0.63	9.25 ± 0.56	9.9 ± 0.51	9.94 ± 0.6
T.Chol (mg/dL)	M	53.7 ± 6.6	52.3 ± 8.03	52.1 ± 10.59	51.4 ± 9.94	49 ± 7.58	46.8 ± 7.05
	F	37.2 ± 6.2	41.8 ± 8.19	36.4 ± 6.4	40.5 ± 8.3	41.4 ± 4.28	38 ± 9.43
Creatinine (mg/dL)	M	0.52 ± 0.04	0.52 ± 0.04	0.5 ± 0.04	0.51 ± 0.03	0.53 ± 0.02	0.49 ± 0.03^*∗*^
	F	0.57 ± 0.05	0.57 ± 0.03	0.56 ± 0.06	0.56 ± 0.05	0.56 ± 0.04	0.59 ± 0.04
Glucose (mg/dL)	M	113.7 ± 15.78	107 ± 9.45	114.6 ± 13.52	109.6 ± 15.75	101.6 ± 23.37	105.4 ± 24.81
	F	99.8 ± 17.14	94.2 ± 17.46	91.8 ± 18.41	94.4 ± 18.01	102 ± 11.6	95.8 ± 22.49
HDL (mg/dL)	M	50.6 ± 6.7	57.3 ± 9.99	58.3 ± 10.52	54.4 ± 9.96	46.8 ± 6.83	47.6 ± 8.32
	F	44.1 ± 7.31	48.5 ± 8.73	45.8 ± 6.55	45.3 ± 6.13	44.4 ± 5.86	42 ± 9.67
LDL (mg/dL)	M	16.2 ± 2.97	16.7 ± 2.91	16.7 ± 3.68	17.6 ± 3.44	33.6 ± 4.22	33.2 ± 2.49
	F	9 ± 2.54	9.3 ± 1.06	9.2 ± 1.93	9 ± 2.62	27.2 ± 1.3	29.4 ± 2.51
Phosphorus (mg/dL)	M	5.43 ± 0.53	5.16 ± 0.38	5.4 ± 0.56	5.43 ± 0.49	5.22 ± 0.36	4.7 ± 0.19^*∗*^
	F	3.98 ± 0.44	4.49 ± 0.52	4.4 ± 0.41	4.17 ± 0.53	3.96 ± 0.63	4.44 ± 0.99
TP (g/dL)	M	6.02 ± 0.26	6.17 ± 0.27	6.26 ± 0.32	6.16 ± 0.29	6.24 ± 0.36	6.32 ± 0.48
	F	6.2 ± 0.54	6.18 ± 0.35	5.94 ± 0.35	6.23 ± 0.55	6.48 ± 0.27	6.5 ± 0.38
Triglyceride (mg/dL)	M	100.5 ± 35.77	108.9 ± 46.44	105.5 ± 29.11	100.7 ± 27.6	98 ± 40.24	101.6 ± 23.2
	F	64.3 ± 17.45	66.8 ± 34.06	65.9 ± 28.18	68.4 ± 24.36	82.4 ± 29.48	78.8 ± 20.79
Urea (mg/dL)	M	48 ± 8.52	51 ± 8.62	49.9 ± 9.89	46.6 ± 11.61	46.2 ± 4.87	42.2 ± 4.87
	F	46.9 ± 5.4	45.2 ± 4.69	44 ± 4.67	44.3 ± 8.68	44.2 ± 6.94	47.6 ± 2.61
Sodium (mmoL/L)	M	139.3 ± 1.35	140.22 ± 1.57	139.86 ± 1.37	138.68 ± 1.45	138.56 ± 0.94	139.46 ± 0.62
	F	138.61 ± 0.24	138.32 ± 1.2	137.74 ± 1.07	139.14 ± 1.78	139.72 ± 1.19	139.54 ± 1.12
Potassium (mmoL/L)	M	4.55 ± 0.26	4.41 ± 0.31	4.36 ± 0.3	4.62 ± 0.38	4.14 ± 0.13	4.34 ± 0.29
	F	4.09 ± 0.3	4.18 ± 0.15	3.94 ± 0.28	4.15 ± 0.3	4.19 ± 0.24	4.09 ± 0.22
Chloride (mmoL/L)	M	108.45 ± 1.3	107.8 ± 1.41	107.98 ± 0.96	109.1 ± 1.15	106.4 ± 0.94	107.06 ± 0.83
	F	109.07 ± 0.97	109.28 ± 0.95	108.67 ± .07	108.28 ± 1.25	107.12 ± 0.64	107.84 ± 0.94

Data are presented as mean ± SD; ^*∗*^ indicates significant changes (*p* < 0.05) compared with the control group. ALP, alkaline phosphatase; ALT, alanine aminotransferase; AST, aspartate aminotransferase; BUN, blood urea nitrogen; HDL, high-density lipoproteins; LDL, low-density lipoproteins; T.Bil, total bilirubin; TP, total protein, T.Chol, total cholesterol.

**Table 5:** Effect of 90-day oral administration of LN18178 on the absolute and relative organ weights of male Wistar rats.

Organs	LN18178 dose (mg/kg·BW)					
	Main groups (*n* = 10)				Reversal groups (*n* = 5)	
	0	625	1250	2500	0	2500
Liver (g)	10.17 ± 1.07	9.89 ± 1.22	9.93 ± 1.21	9.46 ± 1.48	11.45 ± 1.42	11.39 ± 1.27
% of BW	2.78 ± 0.39	2.65 ± 0.27	2.66 ± 0.10	2.56 ± 0.23	2.83 ± 0.44	2.84 ± 0.14
Kidneys (g)	2.35 ± 0.23	2.22 ± 0.29	2.31 ± 0.24	2.36 ± 0.30	2.56 ± 0.32	2.69 ± 0.33
% of BW	0.64 ± 0.05	0.60 ± 0.06	0.62 ± 0.03	0.64 ± 0.06	0.63 ± 0.08	0.67 ± 0.08
Adrenal glands (g)	0.06 ± 0.01	0.06 ± 0.01	0.06 ± 0.01	0.06 ± 0.01	0.05 ± 0.01	0.06 ± 0.00
% of BW	0.02 ± 0.00	0.02 ± 0.00	0.02 ± 0.00	0.02 ± 0.00	0.01 ± 0.00	0.01 ± 0.00
Heart (g)	1.06 ± 0.14	1.08 ± 0.10	1.11 ± 0.10	1.15 ± 0.23	1.21 ± 0.10	1.17 ± 0.08
% of BW	0.29 ± 0.02	0.29 ± 0.02	0.29 ± 0.03	0.31 ± 0.05	0.30 ± 0.03	0.29 ± 0.01
Brain (g)	2.04 ± 0.09	2.00 ± 0.10	2.08 ± 0.19	2.06 ± 0.12	2.01 ± 0.07	2.05 ± 0.16
% of BW	0.56 ± 0.06	0.54 ± 0.03	0.56 ± 0.04	0.56 ± 0.06	0.50 ± 0.03	0.51 ± 0.05
Spleen (g)	0.69 ± 0.11	0.69 ± 0.08	0.64 ± 0.08	0.71 ± 0.12	0.75 ± 0.05	0.72 ± 0.06
% of BW	0.19 ± 0.05	0.19 ± 0.02	0.17 ± 0.02	0.19 ± 0.03	0.19 ± 0.02	0.18 ± 0.02
Thymus (g)	0.42 ± 0.08	0.41 ± 0.06	0.39 ± 0.07	0.44 ± 0.10	0.37 ± 0.03	0.32 ± 0.05
% of BW	0.11 ± 0.02	0.11 ± 0.01	0.10 ± 0.01	0.12 ± 0.03	0.09 ± 0.01	0.08 ± 0.01
Testes (g)	3.72 ± 0.58	3.56 ± 0.30	3.70 ± 0.38	3.58 ± 0.31	3.59 ± 0.18	3.77 ± 0.24
% of BW	1.01 ± 0.14	0.96 ± 0.07	1.00 ± 0.08	1.13 ± 0.50	0.89 ± 0.11	0.94 ± 0.10
Epididymis (g)	1.45 ± 0.10	1.40 ± 0.12	1.45 ± 0.18	1.46 ± 0.15	1.56 ± 0.14	1.68 ± 0.16
% of BW	0.40 ± 0.04	0.38 ± 0.02	0.39 ± 0.03	0.40 ± 0.03	0.39 ± 0.05	0.42 ± 0.03
SV-CG and prostate gland (g)	2.98 ± 0.55	2.78 ± 0.35	2.87 ± 0.34	2.93 ± 0.30	2.96 ± 0.30	3.05 ± 0.52
% of BW	0.81 ± 0.11	0.75 ± 0.08	0.78 ± 0.11	0.80 ± 0.08	0.73 ± 0.08	0.78 ± 0.19
Thyroid with parathyroid (g)	0.04 ± 0.02	0.03 ± 0.01	0.02 ± 0.01^*∗*^	0.02 ± 0.00^*∗*^	0.05 ± 0.02	0.06 ± 0.02
% of BW	0.01 ± 0.01	0.01 ± 0.00	0.01 ± 0.00	0.01 ± 0.00	0.01 ± 0.00	0.01 ± 0.00
Pituitary gland (g)	0.01 ± 0.00	0.01 ± 0.00	0.01 ± 0.00	0.01 ± 0.00	0.01 ± 0.00	0.01 ± 0.00
% of BW	0.00 ± 0.00	0.00 ± 0.00	0.00 ± 0.00	0.00 ± 0.00	0.00 ± 0.00	0.00 ± 0.00

Data are presented as mean ± SD. ^*∗*^ indicates significant changes (*p* < 0.05) compared with the control group. BW, body weight; SV-CG, seminal vesicle and coagulating gland.

**Table 6:** Effect of 90-day oral administration of LN18178 on the absolute and relative organ weights of female Wistar rats.

Organs	LN18178 dose (mg/kg BW)					
	Main groups (*n* = 10)				Reversal groups (*n* = 5)	
	0	625	1250	2500	0	2500
Liver (g)	6.23 ± 0.51	6.41 ± 0.87	6.67 ± 0.57	6.74 ± 0.62	6.81 ± 0.97	7.39 ± 0.55
% of BW	2.76 ± 0.19	2.88 ± 0.37	2.97 ± 0.19	3.01 ± 0.20	2.80 ± 0.38	3.06 ± 0.28
Kidneys (g)	1.48 ± 0.10	1.40 ± 0.14	1.51 ± 0.14	1.49 ± 0.14	1.66 ± 0.08	1.74 ± 0.17
% of BW	0.67 ± 0.03	0.63 ± 0.04	0.67 ± 0.04	0.67 ± 0.05	0.68 ± 0.06	0.72 ± 0.09
Adrenals (g)	0.08 ± 0.01	0.07 ± 0.01	0.16 ± 0.24	0.08 ± 0.02	0.06 ± 0.00	0.07 ± 0.01
% of BW	0.04 ± 0.00	0.03 ± 0.00	0.07 ± 0.11	0.04 ± 0.01	0.03 ± 0.00	0.03 ± 0.00
Heart (g)	0.73 ± 0.07	0.70 ± 0.04	0.75 ± 0.06	0.77 ± 0.09	0.82 ± 0.03	0.82 ± 0.14
% of BW	0.32 ± 0.03	0.32 ± 0.01	0.33 ± 0.01	0.34 ± 0.03	0.33 ± 0.03	0.34 ± 0.06
Brain (g)	1.90 ± 0.08	1.87 ± 0.12	1.89 ± 0.11	1.90 ± 0.15	1.82 ± 0.10	1.89 ± 0.21
% of BW	0.85 ± 0.05	0.84 ± 0.07	0.84 ± 0.06	0.85 ± 0.09	0.75 ± 0.06	0.78 ± 0.07
Spleen (g)	0.50 ± 0.06	0.49 ± 0.06	0.54 ± 0.08	0.49 ± 0.06	0.53 ± 0.03	0.57 ± 0.09
% of BW	0.22 ± 0.03	0.22 ± 0.03	0.24 ± 0.02	0.18 ± 0.03^*∗*^	0.22 ± 0.01	0.24 ± 0.04
Thymus (g)	0.38 ± 0.07	0.34 ± 0.04	0.35 ± 0.09	0.36 ± 0.08	0.28 ± 0.04	0.33 ± 0.04
% of BW	0.17 ± 0.04	0.15 ± 0.01	0.16 ± 0.04	0.16 ± 0.02	0.12 ± 0.02	0.14 ± 0.02
Uterus with cervix	0.65 ± 0.20	0.64 ± 0.17	0.67 ± 0.17	0.83 ± 0.28	0.66 ± 0.18	0.75 ± 0.21
% of BW	0.29 ± 0.09	0.29 ± 0.08	0.30 ± 0.08	0.38 ± 0.14	0.27 ± 0.09	0.31 ± 0.09
Ovaries (g)	0.17 ± 0.05	0.21 ± 0.10	0.18 ± 0.03	0.15 ± 0.04	0.15 ± 0.04	0.17 ± 0.03
% of BW	0.08 ± 0.02	0.10 ± 0.04	0.08 ± 0.02	0.07 ± 0.02	0.06 ± 0.01	0.07 ± 0.01
Thyroid with parathyroid (g)	0.02 ± 0.01	0.02 ± 0.01	0.02 ± 0.00	0.02 ± 0.00	0.02 ± 0.00	0.03 ± 0.00
% of BW	0.01 ± 0.00	0.01 ± 0.00	0.01 ± 0.00	0.01 ± 0.00	0.01 ± 0.00	0.01 ± 0.00
Pituitary (g)	0.01 ± 0.00	0.01 ± 0.00	0.01 ± 0.00	0.01 ± 0.00	0.01 ± 0.00	0.01 ± 0.00
% of BW	0.01 ± 0.00	0.01 ± 0.00	0.01 ± 0.00	0.01 ± 0.00	0.00 ± 0.00	0.00 ± 0.00

Data are presented as mean ± SD. ^*∗*^ indicates significant changes (*p* < 0.05) compared with the control group. BW, body weight.

**Table 7:** Effect of 90-day oral administration of LN18178 on hormones in male and female Wistar rats.

Measurements	Sex	LN18178 dose (mg/kg·BW)					
		Main groups (*n* = 20)				Reversal groups (*n* = 10)	
		0	625	1250	2500	0	2500
T3 (ng/mL)	M	0.42 ± 0.09	0.34 ± 0.07	0.44 ± 0.10	0.40 ± 0.06	0.36 ± 0.11	0.38 ± 0.15
	F	0.39 ± 0.05	0.43 ± 0.09	0.33 ± 0.06	0.44 ± 0.09	0.41 ± 0.07	0.44 ± 0.14
T4 (*μ*g/dL)	M	4.00 ± 0.60	3.62 ± 0.57	3.62 ± 0.54	4.36 ± 0.73	4.20 ± 0.38	4.43 ± 0.70
	F	3.11 ± 0.61	2.74 ± 0.74	3.43 ± 0.77	2.99 ± 0.39	3.46 ± 0.42	3.11 ± 0.32
TSH (ng/mL)	M	1.63 ± 0.22	1.62 ± 0.24	1.57 ± 0.19	1.55 ± 0.17	1.71 ± 0.33	1.62 ± 0.27
	F	1.62 ± 0.19	1.62 ± 0.18	1.56 ± 0.18	1.64 ± 0.26	1.70 ± 0.38	1.65 ± 0.26

Data are presented as mean ± SD. T3, triiodothyronine; T4, thyroxine; TSH, thyroid-stimulating hormone. Each main group (*n* = 20; 10 male and 10 female rats); each reversal group (*n* = 10; 5 male and 5 female rats).

**Table 8:** Summary of histopathological changes in the vital organs of the male and female rats after 90-day oral administration of LN18178.

Organs	Pathological observations	Dose (mg/kg/day BW)			
		0	2500	0	2500
		Male (*n* = 10)		Female (*n* = 10)	
Adrenal glands	Hemorrhage, zona fasciculata	0	0	1 (++)	0
Eyes	Hyperplasia, retina	0	0	1 (+)	0
	Folds, retina	0	0	0	1 (++)
Lungs	Hemorrhage, alveolar	1 (++)	1 (+)	1 (++)	0
	Pigmented macrophage	0	2 (+)	0	0
Stomach	Increased mucosa-associated lymphoid tissue	0	0	1 (+)	0
Duodenum	Yellow pigment	0	1(+)	0	0
Liver	Necrosis	1 (++)	0	0	0
Kidneys	Lymphocyte infiltrate, cortex	1 (++)	1 (++)	0	0
Mesenteric lymph nodes	Pigmented macrophage	0	1 (++)	0	0
Testes	Inflammation	1 (++)	1 (++)	—	—
Epididymis	Inflammation	1 (+)	1 (+++)	—	—

The scores in parentheses +, ++, and +++ indicates minimal, mild, and marked effects, respectively; BW, body weight.

**Table 9:** Summary of observations of *in vitro* bacterial reverse mutation assay in the presence or absence of the metabolic activation.

S9	Treatments	Concn. (*μ*g/plate)	Revertant colony count (mean ± SD)						
			TA97a	TA98	TA100	TA102	TA1535	TA1537	WP2uvrA
+	DMSO^a^	0	114.33 ± 10.79	25.67 ± 2.52	124.67 ± 1.53	322.00 ± 18.33	14.67 ± 0.58	12.00 ± 1.00	13.33 ± 1.53
	LN18178	312.5	103.00 ± 5.29	24.67 ± 1.53	121.67 ± 2.08	314.00 ± 6.24	14.33 ± 1.53	10.67 ± 1.15	11.00 ± 1.00
		625	108.00 ± 3.61	23.00 ± 1.00	121.67 ± 3.06	306.33 ± 2.52	13.00 ± 2.00	11.67 ± 0.58	10.67 ± 1.53
		1250	113.00 ± 3.61	23.67 ± 1.53	120.67 ± 2.52	318.00 ± 3.61	13.00 ± 1.00	10.67 ± 1.53	12.00 ± 1.73
		2500	110.67 ± 6.43	26.00 ± 1.00	123.67 ± 2.52	321.33 ± 2.52	13.00 ± 1.00	12.00 ± 1.00	10.33 ± 1.53
		5000	107.00 ± 4.36	23.67 ± 1.53	121.33 ± 1.53	310.33 ± 3.79	14.33 ± 0.58	11.33 ± 1.15	11.67 ± 1.53
	2-Amino-anthracene^b^	20	1335.00 ± 44.84^*∗*^	1204.67 ± 20.13^*∗*^	1228.33 ± 37.63^*∗*^	2704.00 ± 189.75^*∗*^	1164.00 ± 54.44^*∗*^	200.33 ± 13.20^*∗*^	—
		30	—	—	—	—	—	—	173.67 ± 21.55^*∗*^

−	DMSO^a^	0	121.33 ± 3.51	26.67 ± 2.08	124.67 ± 2.52	235.67 ± 8.50	12.67 ± 0.58	10.00 ± 2.65	12.67 ± 1.53
	LN18178	312.5	118.33 ± 5.51	24.00 ± 1.00	122.67 ± 1.53	229.00 ± 3.00	12.00 ± 1.00	8.67 ± 0.58	10.33 ± 1.53
		625	113.33 ± 3.79	26.00 ± 2.00	121.00 ± 2.00	233.67 ± 4.51	11.33 ± 1.15	9.00 ± 1.00	10.67 ± 0.58
		1250	122.00 ± 1.00	25.33 ± 1.53	121.67 ± 2.52	234.67 ± 5.13	12.00 ± 1.00	9.67 ± 0.58	11.33 ± 2.08
		2500	121.00 ± 2.65	26.00 ± 2.00	123.00 ± 2.00	230.67 ± 2.52	11.33 ± 1.53	9.33 ± 0.58	11.00 ± 1.00
		5000	121.33 ± 1.53	26.33 ± 1.53	122.33 ± 1.53	232.00 ± 3.61	11.33 ± 0.58	9.33 ± 1.53	10.67 ± 1.53
	9-Aminoacridine^b^	50	1335.33 ± 44.74^*∗*^	—	—	—	—	178.33 ± 24.11^*∗*^	—
	Sodium azide^b^	10	—	—	1229.33 ± 36.30^*∗*^	—	1182.33 ± 45.63^*∗*^	—	—
	2-Nitrofluorene^b^	25	—	1274.67 ± 40.61^*∗*^	—	—	—	—	—
	4-NQO^b^	3	—	—	—	—	—	—	208.67 ± 15.53^*∗*^
	Ametycin^b^	0.5	—	—	—	1998.00 ± 26.00^*∗*^	—	—	—

Data are presented as mean ± SD, DMSO, dimethylsulfoxide, NQO, nitro quinoline oxide; ^a^ and ^b^ indicate vehicle and positive controls, respectively, ^*∗*^ indicates significant changes (*p* < 0.05) compared with the control group.

**Table 10:** Observations on chromosomal aberration induced by LN18178.

Treatments	Concn. (*μ*g/mL)	Number of cells scored	Number of structural aberrant cells without gap		
			−S9		+S9
			4 h	22 h	4 h
DMSO^a^	0	300	6	2	2
	250	300	—	2	4
LN18178	500	300	3	2	2
	1000	300	1	3	2
	2000	300	4	—	—
Mitomycin C^b^	0.3	300	33^*∗*^	37^*∗*^	—
Cyclophosphamide^b^	10	300	—	—	28^*∗*^

^a^DMSO, dimethylsulfoxide; vehicle control, ^b^positive controls. ^*∗*^ indicates significant changes (*p* < 0.05) compared with the vehicle control.

**Table 11:** Micronucleated polychromatic erythrocytes (MNPCEs) in LN18178-treated mice bone marrow.

Treatments	Dose (mg/kg.BW)	Number of mice	PCE/TE	% Reduction	% MNPCE
Vehicle control^a^	0	10	46.3	—	0.10 ± 0.07
	500	10	46.2	0.2	0.11 ± 0.05
LN18178	1000	10	46.8	−1.1	0.07 ± 0.06
	2000	10	46.3	0	0.07 ± 0.06
Cyclophosphamide^b^	40	10	41.2	11.2	1.48 ± 0.19^*∗*^

^*∗*^indicates significant changes (*p* < 0.05) compared with the vehicle control. ^a^Distilled water, ^b^positive control; BW, body weight; MNPCE, micronucleated polychromatic erythrocyte; PCE, polychromatic erythrocyte; TE, total erythrocytes. Each group contains 10 animals (5 male and 5 female).

**Table 12:** Cytotoxicity and mutagenicity of LN18178 in mouse lymphoma cells, L5178Y TK^+/−^

Experiment duration	Concentration (*μ*g/mL)	Metabolic activation system	RSG (%)	RCE (%)	RTG (%)	Cloning efficiency (CE_V_)	Cloning efficiency (CE_M_)	Mutant frequency (10^−6 ^cells)	% SC
3 hr	0 (NC)	−	118.20	90.56	107.05	1.00	72.79	73	24
		+	116.22	87.16	101.30	0.97	111.96	118	16
	0 (VC)	−	100.00	100.00	100.00	1.10	95.89	87	17
		+	100.00	100.00	100.00	1.11	108.72	98	15
	62.5	−	80.61	96.73	77.97	1.06	118.59	111	20
		+	79.19	93.96	74.41	1.05	94.38	90	20
	125	−	71.65	96.52	69.16	1.06	97.45	92	26
		+	56.50	88.04	49.74	0.98	102.53	104	21
	250	−	39.71	93.25	37.03	1.03	104.08	105	12
		+	29.59	86.16	25.50	0.96	123.44	129	23
	500	−	19.16	91.98	17.62	1.01	125.91	125	11
		+	12.55	100.82	12.66	1.12	77.41	71	19
	0.1 (NQO)	−	50.17	77.54	38.90	0.85	402.42	472	57
	5 (CPC)	+	30.37	81.56	24.77	0.91	500.45	569	49

24 hr	0 (NC)		115.91	87.34	101.23	0.90	75.78	86	9
	0 (VC)		100.00	100.00	100.00	1.03	102.43	99	21
	15.63		68.75	106.14	72.97	1.10	83.52	76	15
	31.25	−	47.31	102.94	48.70	1.06	139.32	131	20
	62.50		31.99	100.13	32.03	1.03	126.82	123	14
	125.00		15.58	98.49	15.34	1.02	99.06	98	22
	0.1 (NQO)		31.77	79.58	25.28	0.81	681.69	831	65

^*∗*^NC, negative control (water); VC, vehicle control (1%, DMSO); NQO, 4-nitroquinoline-1-oxide; CPC, cyclophosphamide; SC, small colony; RSG, relative suspension growth; RCE, relative cloning efficiency; RTG, relative total growth; CE_V_, cloning efficiency (viable count) plates, CE_M_, cloning efficiency mutant selection plates.

## Data Availability

The complete data used to support the findings of this study are available upon request.

## Abbreviations

BW: Body weight
CPCSEA: Committee for the Purpose of Control and Supervision of Experiments on Animals
CT: Clotting time
DMEM: Dulbecco's modified Eagle medium
DNA: Deoxyribonucleic acid
GLP: Good laboratory practice
HPLC: High-performance liquid chromatography
IAEC: Institutional Animal Ethics Committee
LD_50_: Median lethal dose
MNPCEs: Micronucleated polychromatic erythrocytes
MTT: Methyl-thiazolyl diphenyl-tetrazolium bromide
NCE: Normochromatic erythrocytes
NOAEL: No-observed-adverse-effect level
OECD: Organization for Economic Cooperation and Development
PCEs: Polychromatic erythrocytes
T3: Triiodothyronine
T4: Thyroxin
TSH: Thyroid-stimulating hormone
TFT: Trifluorothymidine.
